# Supplementary material for: Call for emergency action to limit global temperature increases, restore biodiversity, and protect health - Wealthy nations must do much more, much faster
Source: Rev Saude Publica. 2021 Sep 3;55:1ed. doi: 10.11606/s1518-8787.20210550001ed (PMC8425960; doi:10.11606/s1518-8787.20210550001ed)
Supplement: Translation

# Translation: Apelo por ação emergencial para limitar o aumento da temperatura global, restaurar a biodiversidade e proteger a saúde - As nações ricas devem fazer muito mais, muito mais rápido^a^

**Authors:** Lukoye Atwoli, Abdullah H Baqui, Thomas Benfield, Raffaella Bosurgi, Fiona Godlee, Stephen Hancocks, Richard Horton, Laurie Laybourn-Langton, Carlos Augusto Monteiro, Ian Norman, Kirsten Patrick, Nigel Praities, Marcel GM Olde Rikkert, Eric J Rubin, Peush Sahni, Richard Smith, Nicholas J Talley, Sue Turale, Damián Vázquez

**Affiliations:** I Kenya East African Medical Journal. Nairobi, Kenya; II USA Journal of Health, Population and Nutrition. Baltimore, Maryland, USA; III Denmark Danish Medical Journal. Copenhagen, Denmark; IV UK PLOS Medicine. Cambridge, UK; V UK The BMJ. London, UK; VI UK British Dental Journal. London, UK; VII UK The Lancet. London, UK; VIII UK UK Health Alliance on Climate Change. London, UK; IX Brazil Revista de Saúde Pública. São Paulo, SP, Brazil; X UK International Journal of Nursing Studies. London, UK; XI Canada CMAJ. Ottawa, ON, Canada; XII UK Pharmaceutical Journal. London, UK; XIII Netherlands Dutch Journal of Medicine. Amsterdam, Netherlands; XIV USA NEJM. Boston, MA, USA; XV India National Medical Journal of India. New Delhi, Delhi, India; XVI Australia Medical Journal of Australia. Sydney, NSW, Australia; XVII Switzerland International Nursing Review. Geneva, Switzerland; XVIII USA Pan American Journal of Public Health. Washington, DC, USA

A Assembleia Geral da ONU, em setembro de 2021, reunirá os países em um momento crítico para uma ação coletiva de enfrentamento da crise ambiental global. Eles se reunirão novamente na cúpula da biodiversidade em Kunming, na China, e na conferência sobre o clima (COP26) em Glasgow, no Reino Unido. Antes dessas reuniões cruciais, nós, editores de revistas de saúde em todo o mundo, pedimos ações urgentes para manter o aumento médio da temperatura global abaixo de 1,5°C, parar a destruição da natureza e proteger a saúde.

A saúde já está sendo prejudicada pelo aumento da temperatura global e pela destruição do mundo natural, situação para a qual os profissionais de saúde vêm chamando a atenção há décadas^1^. A ciência é inequívoca; um aumento global de 1,5°C acima da média pré-industrial e a perda contínua da biodiversidade trazem danos catastróficos à saúde que serão impossíveis de reverter^2,3^. Apesar da preocupação necessária do mundo com a covid-19, não podemos esperar que a pandemia passe para então reduzirmos rapidamente as emissões.

Refletindo a gravidade do momento, este mesmo editorial está sendo publicado simultaneamente por várias revistas científicas do campo da saúde, em vários países. Estamos unidos em reconhecer que apenas mudanças fundamentais e equitativas nas sociedades reverterão nossa trajetória atual.

Os riscos à saúde de aumentos acima de 1,5°C estão agora bem estabelecidos^2^. De fato, nenhum aumento de temperatura é “seguro”. Nos últimos 20 anos, a mortalidade relacionada ao calor entre pessoas com mais de 65 anos aumentou em mais de 50%^4^. Temperaturas mais altas trouxeram aumento da desidratação e perda da função renal, neoplasias dermatológicas, infecções tropicais, desfechos adversos de saúde mental, complicações na gravidez, alergias e morbimortalidade cardiovascular e pulmonar^5,6^. Os danos afetam desproporcionalmente os mais vulneráveis, inclusive crianças, populações mais velhas, minorias étnicas, comunidades mais pobres e pessoas com problemas de saúde subjacentes^2,4^.

O aquecimento global também está contribuindo para o declínio do potencial de rendimento global dos principais cultivos, reduzido em 1,8–5,6% desde 1981; isso, somado aos efeitos do clima extremo e do esgotamento do solo, está dificultando os esforços para reduzir a desnutrição^4^. Ecossistemas prósperos são essenciais para a saúde humana. A destruição generalizada da natureza, incluindo habitats e espécies, está corroendo a segurança hídrica e alimentar e aumentando a chance de pandemias^3,7,8^.

As consequências da crise ambiental incidem desproporcionalmente sobre os países e comunidades que menos contribuíram para o problema e são menos capazes de mitigar os danos. No entanto, nenhum país, por mais rico que seja, pode se proteger desses impactos. Permitir que as consequências afetem desproporcionalmente os mais vulneráveis irá gerar mais conflitos, insegurança alimentar, deslocamento forçado e doenças zoonóticas — com graves implicações para todos os países e comunidades. Como a pandemia covid-19 está nos ensinando, somos globalmente tão fortes quanto nosso membro mais fraco.

Aumentos acima de 1,5°C aumentam a chance de atingir pontos de inflexão em sistemas naturais que poderiam colocar o mundo em um estado agudo de instabilidade. Isso prejudicaria criticamente nossa capacidade de mitigar danos e evitar mudanças ambientais catastróficas e descontroladas^9,10^.

## Metas Globais Não São Suficientes

Encorajadoramente, muitos governos, instituições financeiras e empresas estão estabelecendo metas para atingir zero emissões líquidas, incluindo metas para 2030. O custo da energia renovável está caindo rapidamente. Muitos países pretendem proteger pelo menos 30% das terras e oceanos do mundo até 2030^11^.

Essas promessas não são suficientes. Metas são fáceis de definir e difíceis de alcançar. Elas ainda precisam ser combinadas com planos confiáveis de curto e longo prazo para acelerar tecnologias mais limpas e transformar sociedades. Planos de redução de emissões não incorporam adequadamente considerações de saúde^12^. Há uma crescente preocupação de que o aumento da temperatura acima de 1,5°C está começando a ser visto como inevitável, ou mesmo aceitável, por membros poderosos da comunidade global^13^. Além disso, as estratégias atuais para reduzir as emissões líquidas a zero até meados do século presumem que o mundo adquirirá grandes capacidades para remover gases de efeito estufa da atmosfera^14,15^.

Uma ação insuficiente significa que os aumentos de temperatura provavelmente serão bem superiores a 2°C^16^, um resultado catastrófico para a saúde e a estabilidade ambiental. Algo de suma importância é que a destruição da natureza não tem paridade de estima com o elemento climático da crise e nenhuma meta global para restaurar a perda de biodiversidade até 2020 foi alcançada^17^. Essa é uma crise ambiental global^18^.

Os profissionais de saúde estão unidos com cientistas ambientais, empresas e muitos outros atores na rejeição de que esse resultado seja inevitável. Nós podemos e devemos fazer mais agora — em Glasgow e Kunming — e nos anos imediatamente seguintes. Unimo-nos a profissionais de saúde em todo o mundo que já apoiaram pedidos de ação rápida^1,19^.

A equidade deve estar no centro da resposta global. Contribuir com uma parte justa para o esforço global significa que os compromissos de redução devem levar em conta a contribuição histórica cumulativa que cada país tem feito às emissões, bem como suas emissões atuais e capacidade de resposta. Os países mais ricos terão que diminuir as emissões mais rapidamente, reduzindo até 2030 mais do que o proposto atualmente^20,21^ e atingindo zero emissões líquidas antes de 2050. Metas semelhantes e ações emergenciais são necessárias para prevenir a perda da biodiversidade e a destruição mais ampla do mundo natural.

Para atingir esses objetivos, os governos devem fazer mudanças fundamentais na forma como nossas sociedades e economias estão organizadas e como vivemos. A estratégia atual de incentivar os mercados a trocar tecnologias sujas por outras mais limpas não é suficiente. Os governos devem intervir para apoiar o redesenho de sistemas de transporte, cidades, produção e distribuição de alimentos, mercados para aplicações financeiras, sistemas de saúde e muito mais. A coordenação global é necessária para garantir que a corrida por tecnologias mais limpas não venha ao custo de mais destruição ambiental e exploração humana.

Muitos governos enfrentaram a ameaça da pandemia de covid-19 com financiamento sem precedentes. A crise ambiental exige uma resposta de emergência semelhante. Serão necessários grandes investimentos, muito além do que está sendo considerado ou entregue em qualquer lugar do mundo. Mas esses investimentos produzirão enormes resultados positivos para a saúde e a economia. Estes incluem empregos de alta qualidade, redução da poluição do ar, aumento da atividade física e melhores moradias e dieta. Só uma melhor qualidade do ar já resultaria em benefícios para a saúde que compensariam facilmente os custos globais da redução de emissões^22^.

Essas medidas também serão positivas para os determinantes sociais e econômicos da saúde, cujas situações precárias podem ter tornado as populações mais vulneráveis à pandemia de covid-19^23^. Entretanto, as mudanças não podem ser alcançadas por meio de um retorno às políticas de austeridade prejudiciais ou na vigência das grandes desigualdades de riqueza e poder intra- e interpaíses.

## A Cooperação Depende de Nações Ricas Fazerem Mais

Em particular, os países que criaram desproporcionalmente a crise ambiental devem fazer mais para apoiar países de baixa e média renda para construir sociedades mais limpas, saudáveis e resilientes. Os países de alta renda devem cumprir e ir além de seu compromisso pendente de fornecer US$ 100 bilhões por ano, compensando qualquer déficit em 2020 e aumentando as contribuições até e para além de 2025. O financiamento deve ser dividido igualmente entre mitigação e adaptação, incluindo melhoria na resiliência dos sistemas de saúde.

O financiamento deve ser por meio de subvenções ao invés de empréstimos, de modo a construir capacidades locais e verdadeiramente capacitar comunidades, devendo também ser acompanhado do perdão de grandes dívidas, que restringem a autonomia de tantos países de baixa renda. Fundos adicionais devem ser destinados a compensar perdas e danos inevitáveis causados pelas consequências da crise ambiental.

Como profissionais de saúde, devemos fazer todo o possível para ajudar na transição para um mundo sustentável, mais justo, resiliente e saudável. Além de agir para reduzir os danos causados pela crise ambiental, devemos contribuir proativamente para a prevenção global de mais danos e agir sobre as causas básicas da crise. Devemos responsabilizar os líderes globais e continuar a educar os demais sobre os riscos dessa crise à saúde. Devemos trabalhar juntos para alcançar sistemas de saúde ambientalmente sustentáveis antes de 2040, reconhecendo que isso implicará mudanças em práticas clínicas. As instituições de saúde já desinvestiram mais de US$ 42 bilhões em ativos de combustíveis fósseis; outras devem se juntar a elas^4^.

A maior ameaça à saúde pública global é o fracasso contínuo dos líderes mundiais em manter o aumento da temperatura global abaixo de 1,5°C e restaurar a natureza. Mudanças urgentes em toda a sociedade devem ser feitas e levarão a um mundo mais justo e saudável. Nós, como editores de revistas de saúde, fazemos um apelo aos governantes e outros líderes para agirem, marcando 2021 como o ano em que o mundo finalmente muda de rumo.
